# Supplementary material for: Insights on the structure and stability of Licanantase: a trimeric acid-stable coiled-coil lipoprotein from Acidithiobacillus thiooxidans
Source: PeerJ. 2014 Aug 5;2:e457. doi: 10.7717/peerj.457 (PMC4137666; doi:10.7717/peerj.457)
Supplement: Figure S1 — Mean percentage of structural properties during last 50 ns in each REMD simulation, Lic-80 (a, d), Lpp-58 (b, e) and Ala-14 (c, f). Color coding: intermonomer H-bonds (black); intermonomers salt bridges (red); main chain H-bonds (orange); hydrophobic contacts (green) and alpha helix (blue). [file peerj-02-457-s001.pdf]

Supplementary Figures

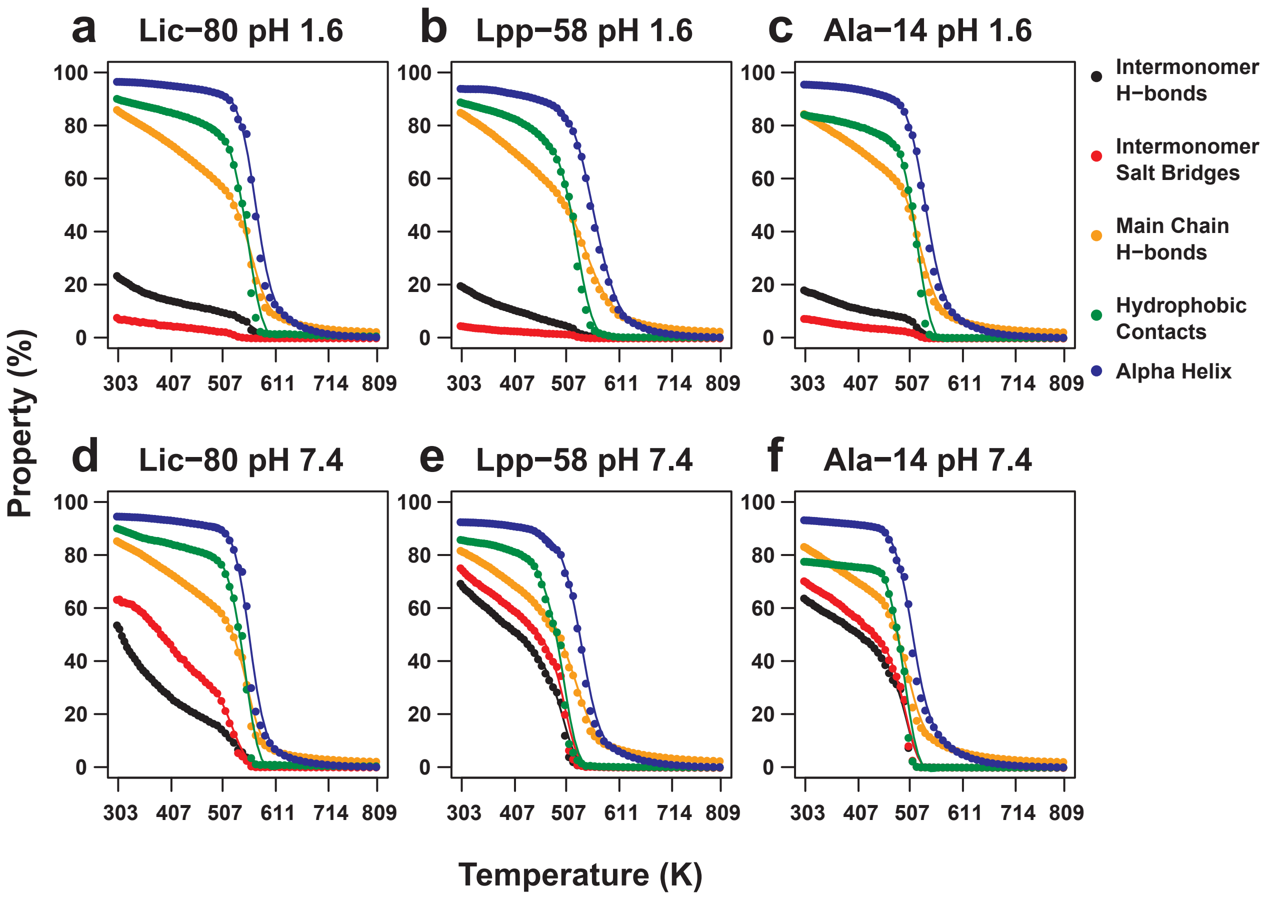

Figure S1 - Mean percentage of structural properties versus temperature.
Mean percentage of structural properties during last 50 ns in each REMD simulation, Lic-80 (a,d), Lpp-58 (b, e) and Ala-14 (c, f). Color coding: Intermonomer H-bonds (Black); Intermonomers Salt Bridges (Red); Main Chain H-bonds (Orange); Hydrophobic Contacts (Green) and Alpha Helix (Blue).
